# *FAM5C* Contributes to Aggressive Periodontitis

**DOI:** 10.1371/journal.pone.0010053

**Published:** 2010-04-07

**Authors:** Flavia M. Carvalho, Eduardo M. B. Tinoco, Kathleen Deeley, Poliana M. Duarte, Marcelo Faveri, Marcelo R. Marques, Adriana C. Mendonça, Xiaojing Wang, Karen Cuenco, Renato Menezes, Gustavo P. Garlet, Alexandre R. Vieira

**Affiliations:** 1 Department of Genetics, Institute of Biology, Federal University of Rio de Janeiro, Rio de Janeiro, Rio de Janeiro, Brazil; 2 Department of Periodontology, School of Dentistry, Rio de Janeiro State University, Rio de Janeiro, Rio de Janeiro, Brazil; 3 Postgraduate Program in Dentistry, School of Health Sciences, UNIGRANRIO, Duque de Caxias, Rio de Janeiro, Brazil; 4 Department of Oral Biology, University of Pittsburgh, Pittsburgh, Pennsylvania, United States of America; 5 Department of Pediatric Dentistry, University of Pittsburgh, Pittsburgh, Pennsylvania, United States of America; 6 Center for Craniofacial and Dental Genetics, School of Dental Medicine, University of Pittsburgh, Pittsburgh, Pennsylvania, United States of America; 7 Department of Human Genetics, Graduate School of Public Health, University of Pittsburgh, Pittsburgh, Pennsylvania, United States of America; 8 Clinical and Translational Sciences Institute, University of Pittsburgh, Pittsburgh, Pennsylvania, United States of America; 9 Department of Periodontics, Dental Research Division, Guarulhos University, Guarulhos, São Paulo, Brazil; 10 Department of Morphology, Division of Histology, School of Dentistry at Piracicaba, University of Campinas, Piracicaba, São Paulo, Brazil; 11 Department of Biological Sciences, School of Dentistry of Bauru, São Paulo University - FOB/USP, Bauru, São Paulo, Brazil; Institute of Infectious Disease and Molecular Medicine, South Africa

## Abstract

Aggressive periodontitis is characterized by a rapid and severe periodontal destruction in young systemically healthy subjects. A greater prevalence is reported in Africans and African descendent groups than in Caucasians and Hispanics. We first fine mapped the interval 1q24.2 to 1q31.3 suggested as containing an aggressive periodontitis locus. Three hundred and eighty-nine subjects from 55 pedigrees were studied. Saliva samples were collected from all subjects, and DNA was extracted. Twenty-one single nucleotide polymorphisms were selected and analyzed by standard polymerase chain reaction using TaqMan chemistry. Non-parametric linkage and transmission distortion analyses were performed. Although linkage results were negative, statistically significant association between two markers, rs1935881 and rs1342913, in the *FAM5C* gene and aggressive periodontitis (p = 0.03) was found. Haplotype analysis showed an association between aggressive periodontitis and the haplotype A-G (rs1935881-rs1342913; p = 0.009). Sequence analysis of *FAM5C* coding regions did not disclose any mutations, but two variants in conserved intronic regions of *FAM5C*, rs57694932 and rs10494634, were found. However, these two variants are not associated with aggressive periodontitis. Secondly, we investigated the pattern of *FAM5C* expression in aggressive periodontitis lesions and its possible correlations with inflammatory/immunological factors and pathogens commonly associated with periodontal diseases. *FAM5C* mRNA expression was significantly higher in diseased versus healthy sites, and was found to be correlated to the *IL-1β*, *IL-17A*, *IL-4* and *RANKL* mRNA levels. No correlations were found between *FAM5C* levels and the presence and load of red complex periodontopathogens or *Aggregatibacter actinomycetemcomitans*. This study provides evidence that *FAM5C* contributes to aggressive periodontitis.

## Introduction

Aggressive periodontitis is characterized by a rapid and severe periodontal destruction in young systemically healthy subjects, and can be subdivided into localized and generalized forms according to the extension of the periodontal destruction [1]. Epidemiological surveys have shown that the prevalence of aggressive periodontitis varies among ethnic groups, regions and countries, and may range from 0.1% to 15% [2], [3]. A greater prevalence is reported in Africans and African descendent groups than in Caucasians and Hispanics [4], [5].

There are many reports in the literature describing families with multiple aggressive periodontitis affected individuals, suggesting familial aggregation [6]–[8]. Several research groups have used segregation analysis to determine the likely mode of inheritance for this trait. The patterns of disease in these families have led investigators to postulate both dominant and recessive modes of Mendelian inheritance for aggressive periodontitis [9]–[11]. Segregation analysis that included the families in the present study suggested an excessive disease transmission from heterozygous parents. This model provides support for the hypothesis that a few loci, each one with relatively small effects, contribute to aggressive periodontitis, with or without interaction with environmental factors [12].

Candidate gene approaches have been used to study aggressive periodontitis, but the results so far are very diverse and conflicting [13], [14]. A case-control genome wide association study suggested a role for *GLT6D1* in aggressive periodontitis in Germans [15]. One linkage study in African American families [16] showed that aggressive periodontitis is linked to the marker D1S492, located on chromosome 1q. A susceptibility locus for aggressive periodontitis was determined between the markers D1S196 and D1S533. This region of chromosome 1 (from base pair 165,770,752 to base pair 192,424,848) includes the cytogenetic regions from 1q24.2 to 1q31.3. In this study, we first investigated this chromosomal region for genetic variants that contribute to aggressive periodontitis in a clinically well-characterized group of families, several of African descent (Table 1), segregating this condition. The hypothesis of this study is that genetic variation located between 1q24.2 to 1q31.3 contributes to aggressive periodontitis. Since the present genetic studies provide evidence that *FAM5C* gene contributes to aggressive periodontitis, we also investigated the pattern of *FAM5C* expression in periodontal lesions and its possible correlations with inflammatory/immunological factors and pathogens commonly associated with periodontal diseases in a second population presenting aggressive periodontitis, compared to periodontally-healthy controls.

**Table 1:** Ethnic background of the families and number of individuals by affection status and gender in 55 families with at least a proband affected with aggressive periodontitis and average age of the probands.

Family Characteristic	N (%)
**African descent families**	**38 (69%)**
**Affected individuals**	**132 (34%)**
**Probands**	**55**
Male	18
Female	37
Average age of the probands (minimum-maximum)	31.1 years (16–40 years)
**Relatives**	**77**
Male	26
Female	51
**Unaffected individuals**	**193 (49.6%)**
Male	90
Female	103
**Unknown**	**64 (16.4%)**
Male	36
Female	28
**Total**	**389 (100%)**

## Results

### Genetic results

All markers studies (Table 2) were in Hardy-Weinberg equilibrium (data not shown). Non-parametric linkage analysis showed no linkage between genetic markers in 1q24.2-1q31.3 and aggressive periodontitis (Table 3 [48]). Association could be seen between aggressive periodontitis and markers in *FAM5C*, rs1935881 and rs1342913. Both the A allele (common allele) of marker rs1935881 and the G allele (rare allele) of marker rs1342913 were observed to be over-transmitted among cases (p = 0.03 for both, complete results in Table S3). The results of PLINK also suggested an association between aggressive periodontitis and the same marker alleles: most common allele A of marker rs1935881 (OR = 0.50, 95% CI 0.15–1.66, p = 0.07) and rare allele G of marker rs1342913 (OR = 3.2, 95% CI 1.17–8.73, p = 0.03). No linkage disequilibrium was apparent between these two markers (Table S4). Haplotype analysis also showed an association between the haplotype A–G (rs1935881-rs1342913; p = 0.009) and aggressive periodontitis (Table 4). Additional haplotypes including these two markers also had suggestive association results (Table S5).

**Table 2:** Genetic variants studied.

SNP	Position	Gene	Region	Change
rs366839	187,825,289	----	----	AG
rs463228	187,828,998	----	----	AG
rs2208921	187,947,000	----	----	AG
rs12132519	187,997,671	----	----	AG
rs1935885	188,314,448	----	----	AG
rs1935881*	188,333,009	*FAM5C*	3′	GA
rs35296429*	188,333,533	*FAM5C*	3′	-/A
rs1053081*	188,333,608	*FAM5C*	3′	AG
rs35481069*	188,334,216	*FAM5C*	exon 8	AC (K619T)
rs34739035*	188,334,578	*FAM5C*	exon 8	AC (K498N)
rs34098782*	188,334,831	*FAM5C*	exon 8	GA (G414D)
rs10800889	188,341,501	*FAM5C*	intron	AG
rs1342913	188,387,648	*FAM5C*	intron	AG
rs4633293	188,461,532	*FAM5C*	intron	AG
rs12140456	188,479,488	*FAM5C*	intron	CG
rs57694932#	188,705,935	*FAM5C*	intron	AG
rs10494634#	188,706,091	*FAM5C*	intron	AT
rs61818811*	188,713,220	*FAM5C*	5′	AC
rs1377924	189,425,718	----	----	CG
rs2061018	189,429,170	----	----	AT
rs7526348	190,390,334	----	----	AG
rs1175111	190,472,655	----	----	AG
rs1175152	190,479,859	----	----	AG

*FAM5C  = * family with similarity 5, member C;

* Variants included after the first association results;

# Variants found during sequencing of highly conserved intronic regions.

**Table 3:** Results of non-parametric linkage analysis.

Marker	Position (cM)	Z	p value	Delta	Logarithm of Odds §	p value
*	Minimum	−4.96	1	−0.188	−1.64	1
	Maximum	9.6	0	0.707	7.71	0
rs366839	42.129	−0.17	0.6	−0.061	−0.01	0.6
rs463228	42.130	−0.17	0.6	−0.061	−0.01	0.6
rs2208921	42.168	−0.09	0.5	−0.031	0	0.5
rs12132519	42.184	−0.06	0.5	−0.021	0	0.5
rs1935885	42.284	−0.07	0.5	−0.024	0	0.5
rs10800889	42.293	−0.1	0.5	−0.033	0	0.5
rs1342913	42.307	0.28	0.4	0.085	0.02	0.4
rs4633293	42.331	0.43	0.3	0.134	0.06	0.3
rs12140456	42.337	0.43	0.3	0.134	0.06	0.3
rs1377924	42.630	0.28	0.4	0.099	0.03	0.4
rs2061018	42.632	0.43	0.3	0.151	0.06	0.3
rs7526348	42.906	0.11	0.5	0.038	0	0.4
rs1175111	42.927	0.1	0.5	0.035	0	0.5
rs1175152	42.929	0.1	0.5	0.034	0	0.5

* The first two lines indicate the maximum possible scores for this dataset. These are followed by analysis results at each location: cM position, Z score, p-value assuming normal approximation, delta [48], logarithm of odds score [48], and p-value [48].

§ Positive non-parametric logarithm of odds score indicates excess allele sharing among affected individuals. A negative non-parametric logarithm of odds score indicates less than expected allele sharing among these groups of individuals.

**Table 4:** Haplotype results for two, three and four-marker windows in *FAM5C*.

	Haplotype Analysis Results				
FAM5C	rs1935881	rs1342913	rs57694932	rs10494634	Haplotype
					Frequency
p value	0.03	0.03	0.36	0.94	*f*
Sliding Windows					
2 windows	0.009				0.326
		0.03			0.647
			0.07		0.009
3 windows	0.02				0.109
		0.05			0.107
4 windows	0.08				0.110

**Figure 1:**
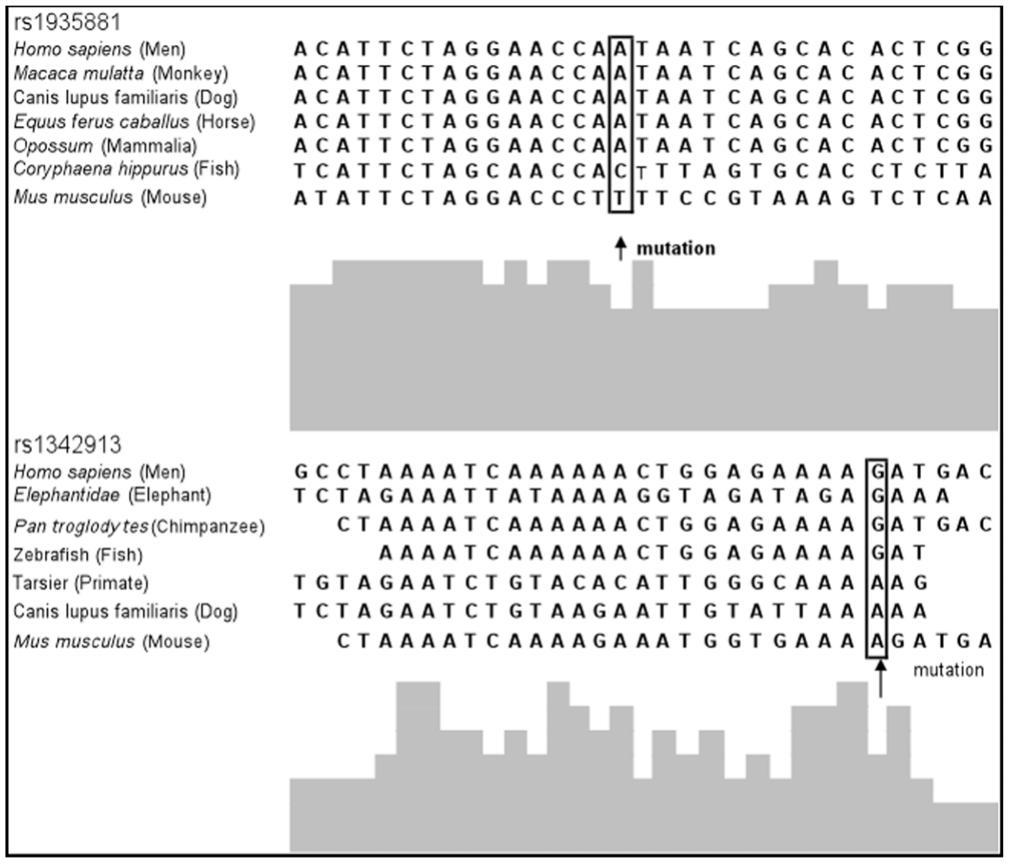
Multispecies sequence comparisons. Multispecies sequence comparison of variant sites (indicated by arrows) in *FAM5C* associated with aggressive periodontitis.

The rs1935881 wild type allele A is conserved in several mammals, while the G allele of rs1342913 is conserved back to zebrafish (Figure 1). The TRANSFAC program predicted the presence of transcription factors in the binding-sites of rs1935881 and rs1342913 (Table S6).

Sequencing of *FAM5C* coding regions did not disclose any etiologic mutations. Two variants were found in highly conserved intronic regions of *FAM5C* gene: rs57694932 and rs10494634. TRANSFAC predicted changes in binding site affinity with these variants (Table S6). These two variants were genotyped in the entire population but they did not show an association with aggressive peridontitis (Tables 3 and S7). They are in moderate linkage disequilibrium (*D*' = 0.655) with the two markers associated with aggressive periodontitis (rs1935881 and rs1342913), which suggests they do not explain the association observed.

The genome wide scan of the two large families not linked to chromosome 1 yielded suggestive results for an association with markers in chromosomes 2q21.2-q37.3, 3p24.2-p24.1, 5p15.2-q33.3, 6p12.3-q12, and 18q12.3q21.2 (p = 0.0009 for all associated markers in these loci; Table 5).

**Table 5:** List of markers associated with aggressive periodontitis in the genome wide scan analysis.

Chromosome 2	Chromosome 3	Chromosome 5	Chromosome 6	Chromosome 18
rs13402622	rs9833191	rs2578619	rs1480617	rs2085796
rs13388210	rs4858608	rs2548552	rs1525354	rs1865555
rs12052971	rs3935025	rs1379544	rs10948618	rs633667
rs10177619	rs2196427	rs7705454	rs4715425	rs11082925
rs6755528	rs3951794	rs1368329	rs9382239	rs12457182
rs6437372	rs6801153	rs11167472	rs513041	rs9320010
rs708078	rs12635000	rs919221	rs2478878	rs188918
rs10173407	rs6774513	rs2548554	rs9381981	rs17800754
rs13029625		rs1382305	rs9357777	rs10502903
rs908265		rs919222	rs11963528	rs9965852
rs6431472		rs10066281	rs7764904	rs8085750
rs6741220		rs2614119	rs2753070	rs1623892
rs1996286		rs17113771	rs2677024	rs1800640
rs4312490		rs10070224	rs824383	rs2919451
rs6749707		rs2244960	rs9296812	rs17785419
rs10460245		rs13181236	rs659446	rs8085360
rs4571012		rs6579746	rs17625497	rs1787614
rs2060127		rs9688110	rs2268855	rs12606093
rs4675792		rs7715047	rs12662737	rs6507852
rs7577417		rs3097779	rs2297985	rs9947627
rs13016717		rs2910263	rs2677023	rs17749350
rs2645778		rs1025260	rs9381454	rs748317
rs4663247		rs286958	rs9474972	rs4613156
rs4398270		rs4566790	rs4094394	rs1787292
rs1822882		rs10038971	rs6929426	rs7235757
rs10166257		rs10796	rs1723527	rs9965625
rs16845023		rs1461240	rs1779758	rs323118
rs13009175		rs778825		rs2969931
rs13032395		rs699113		rs9946886
		rs184586		rs9952398
		rs2244964		rs1800639
		rs10064971		rs9965170
		rs10052410		rs8097738
		rs6883565		rs628531
		rs890832		rs1504504
		rs2548553		rs17707448
		rs6874995		rs627697
		rs6875111		rs1787613
		rs11741184		rs1787606
		rs4702684		rs669350
		rs2662532		rs12456253
		rs183495		rs9304344
		rs2292267		rs533064
				rs1442076
				rs12457104

### Gene expression results

**Figure 2:**
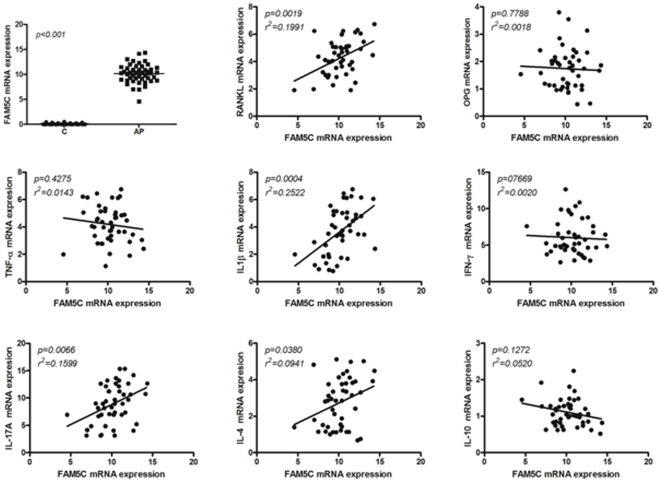
Summary of gene expression results. *FAM5C* expression is significantly higher in diseased tissues. In addition, *FAM5C* mRNA levels were positively correlated with *IL-1β*, *IL-17A*, *IL-4*, and *RANKL* expression, while no correlations were found with *TNF-α*, *IFN-γ*, *IL-10*, and OPG. C  =  periodontally-healthy controls; AP  =  aggressive periodontitis cases.

**Figure 3:**
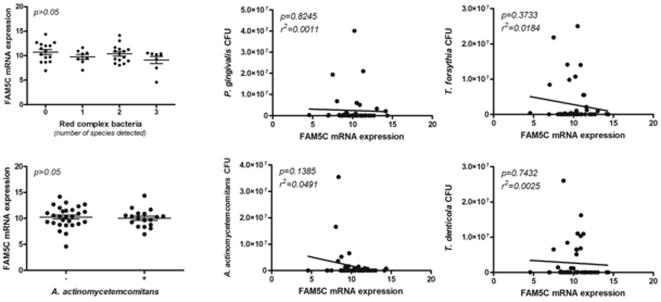
Summary of bacterial DNA quantification results. For red complex bacteria, “0” indicates sites with no bacteria, “1” indicates the detection of one species, “2” indicates the detection of two species, and “3” indicates the detection of three species. *FAM5C* mRNA levels were not associated with the presence or load of red complex periodontopathogens or *Aggregatibacter actinomycetemcomitans*.

In order to support the potential association of *FAM5C* with aggressive periodontitis pathogenesis, we next investigated its expression in diseased versus healthy tissues. Our data demonstrate (Figure 2) that *FAM5C* expression was significantly higher in diseased tissues (p<0.001). In addition, *FAM5C* mRNA levels were positively correlated with *IL-1β* (p = 0.0004, r^2^ = 0.2522), *IL-17A* (p = 0.0066, r^2^ = 0.1599), *IL-4* (p = 0.0380, r^2^ = 0.0941), and *RANKL* (p = 0.0019, r^2^ = 0.1991) expression, while no correlations were found with *TNF-α* (p = 0.4275, r^2^ = 0.0143), *IFN-γ* (p = 07669, r^2^ = 0.0020), *IL-10* (p = 0.1272, r^2^ = 0.0520), and OPG (p = 0.7788, r^2^ = 0.0018). *FAM5C* mRNA levels were not associated with the presence or load of red complex periodontal pathogens or *Aggregatibacter actinomycetemcomitans* (p>0.05; Figure 3).

## Discussion

Aggressive periodontitis is a group of infrequent types of periodontal diseases with rapid attachment loss and bone destruction initiated at a young age. Though a variety of factors, such as microbial, environmental, behavioral and systemic disease, are suggested to influence the risk of aggressive periodontitis, an individual genetic profile is a crucial factor influencing their systemic or host response-related risk [17], [18]. This is the first report that provides evidence of an association between variation in *FAM5C* and aggressive periodontitis. Our work supports the initial findings of linkage [16] between chromosome 1q and aggressive periodontitis.

The family-based study design that we used is robust to problems resulting from population admixture or stratification [19]. Brazil is a trihybrid population of Native Indians, Caucasians with Portuguese ancestry and Africans [20]. The last National Research for Sample of Domiciles census in Rio de Janeiro revealed that in this city 53.4% are white, 46.1% are black, and 0.5% are Asian or Amerindian [21]. Table 1 describes additional demographic variables of the families studied.

We found evidence of association between aggressive periodontitis and *FAM5C*, but not linkage. Since marker allele-disease association and linkage between a disease locus and a marker locus are two different events, linkage without evidence of association and association without evidence of linkage are possible observations [22]. In linkage analysis, we take advantage of the process of forming new allelic combinations (recombination) to identify loci that are linked to the disease. One can argue that these alleles are necessary for the disease to happen. However, an association can exist if the disease-causing variants are in linkage disequilibrium with the associated marker/locus. An association can also exist if the associated genetic marker is a susceptibility locus that increases the probability of developing the disease. By themselves, these alleles are not sufficient for disease manifestation. If the linkage disequilibrium hypothesis is correct, there will be evidence for linkage. If the susceptibility locus hypothesis is correct, there may be strong evidence against linkage [22].

The *FAM5C* gene (NM199051-1, Gene ID:339479) is located on chromosome 1q31.1, comprises eight exons and encodes a protein of 766 amino acids named FAM5C (family with sequence similarity 5, member C; aliases BRINP3, DBCCR1L, RP11-445K1.1). *FAM5C* was originally identified in the mouse brain as a gene that is induced by bone morphogenic protein and retinoic acid signaling [23]. Importantly, FAM5C is localized in the mitochondria and that over-expression of this molecule leads to increased proliferation, migration, and invasion of non-tumorogenic pituitary cells [24], a phenotype relevant to the cellular changes of smooth muscle cells that are associated with the formation and vulnerability of an atherosclerotic plaque [25], [26]. *FAM5C* alleles are also implicated in the risk of myocardial infarction [27]. Through complex signaling cascades, mitochondria have the ability to activate multiple pathways that modulate both cell proliferation and, inversely, promote cell arrest and programmed cell death [28], all phenomena relevant in the pathogenesis of periodontal diseases.

Our exploratory genome wide scan analysis unveiled new candidate loci for aggressive periodontitis. The regions on chromosomes 2, 3, 5, 6, and 18 included many associated markers (Table 5) and spanned over large segments, and included several hundred genes but fine-mapping approaches such as the one used in this study can considerably reduce the time and cost effort to study these loci. Out of the most studied genes in aggressive periodontitis [*IL1-A* and *IL1-B* (2q14), *IL-4* (5q31.1), *IL-10* (1q31-q32), *FcγRIIa*, *FcγRIIb*, and *FcγRIIIb* (1q23), and *TNFA* (6p21.3)], *IL-4* and *TNFA* map in the intervals with suggestive association results. *IL-10* maps in the interval analyzed in the present studied (1q24.2 to 1q31.3) and *FcγRIIa*, *FcγRIIb*, and *FcγRIIIb* are just outside of it.

Interestingly, this preliminary genome wide scan analysis did not suggest linkage to 9q34.3. This locus was recently shown to be associated with aggressive periodontitis in Germans [15]. Since the families studied here are from a distinct geographic location, it is possible that the role of *GLT6D1* in 9q34.3 in these families is less pronounced. Future investigations in our study population include replication of the German genome wide scan finding.

Since literature data is scarce to suggest a mechanism linking FAM5C to the pathogenesis of aggressive periodontitis, we next investigated its pattern of expression in periodontal lesion and possible correlations with inflammatory/immunological and microbial factors classically associated with the periodontitis outcome. *FAM5C* expression was found to be significantly higher in disease tissues, and to present a slight but significant correlation with *IL-1β*, *IL-17A*, *IL-4* and *RANKL* expressions (Figure 2). The pro-inflammatory cytokine IL-1β has been classically associated with inflammatory cell influx and osteoclastogenesis in the periodontal environment [29], and a similar role for IL-17A was recently suggested [30]. Interestingly, both cytokines are positive regulators of *RANKL* expression, the master regulator of osteoclasts differentiation and activation, which is thought to account for alveolar bone loss throughout the periodontal disease process [31]. Conversely, IL-4 was described as an inhibitor of *RANKL* expression, but in certain conditions may increase osteoclast activity [32]. While some studies suggest a possible destructive role for IL-4 in both chronic and aggressive periodontitis [33], [34], other studies suggest that this cytokine has a protective role against tissue destruction [35], [36]. Therefore, it is possible to suppose that FAM5C may somehow modulate/interfere in cytokine network in diseased periodontal tissues, and consequently impact disease outcome. Interestingly, while destructive cytokine expression have been linked to the presence of classic periodontopathogens [33], *FAM5C* mRNA levels were not associated with the presence or load of red complex periodontopathogens or *Aggregatibacter actinomycetemcomitans*, reinforcing the putative strong genetic control of its expression in periodontal tissues.

In summary, this study provides evidence that variation in *FAM5C* might contribute to aggressive periodontitis, and that the markers rs1935881 and rs1342913 are candidate functional variants (based on multispecies nucleotide sequence comparisons and electronic transcription binding site predictions - Figure 1 and Table S6) or are in linkage disequilibrium with still unknown disease-predisposing alleles. Future work will investigate if expression profiles of FAM5C are associated with genetic variation in the gene.

## Materials and Methods

### Subjects (Genetic Studies)

Three hundred and seventy-one subjects from 54 pedigrees (75 nuclear mother-father-affected child) were recruited at the Periodontology Department at the Rio de Janeiro State University (Rio de Janeiro, RJ, Brazil), and UNIGRANRIO (Duque de Caxias, RJ, Brazil) (Figure S1). One additional family was recruited at Guarulhos University (Guarulhos, SP, Brazil) and included father, mother and sixteen offspring (Figure S2). All subjects were of Brazilian descent. The protocol for the study was reviewed and approved by the Ethics Committee of the Rio de Janeiro State University, Guarulhos University, and University of Pittsburgh, and written informed consent was obtained from all individuals prior any research activity. Aggressive periodontitis were diagnosed according to the 1999 international classification of periodontal diseases [1] and positive individuals were assigned as affected. If individuals were edentulous and reported having lost all their teeth at young age (before 35 years), for no obvious reasons such as trauma or extensive cavities, this was recognized as a potential indicator that they started as an aggressive periodontitis case and we also designated them as affected. In addition, the following information was collected by the same examiner from all probands and family members: affection status, gender, age, family relationship and ethnicity, cigarette smoking habits, current medications taken and general health status. In addition, clinical data (pocket probing depth and clinical attachment level) and radiological examinations were collected from all participants. Individuals with co-existing morbidities (e.g. diabetes) or smokers were not defined as affected to minimize the risk of inadvertently including chronic periodontitis in the analysis.

### Isolation of genomic DNA

Saliva samples were collected from all of the 389 individuals with Oragene™ DNA Self-Collection Kit (DNA Genotek Inc., Kanata, ON, Canada). The DNA was extracted using the protocol for manual purification of DNA from 0.5 mL of Oragene™/saliva. The DNA integrity was checked and quantified using the absolute quantification in real-time PCR as suggested by Applied Biosystems (Foster City, CA, USA).

### Selection of single nucleotide polymorphisms (SNPs)

The region between markers D1S196 and D1S533 on chromosome 1(1q24.2-1q31.3), covering about 26 million base pairs, was studied using the data from the International HapMap Project [37] and the University of California Santa Cruz Genome Bioinformatics, and viewed through the software Haploview [38]. Based on pairwise linkage disequilibrium, haplotype block structures, and structure of genes, we identified the 14 most informative single nucleotide polymorphisms in the region (Table 2).

### Genotyping

Polymerase chain reactions [39] with TaqMan chemistry (Applied Biosystems, Foster City, CA, USA) [40] held in total 3 µL/reaction were used for genotyping all selected markers in a PTC-225 tetrad thermocycler (Peltier Thermal Cycler, Bio-Rad Life Sciences, Corston, UK).

### Subjects (Gene expression studies)

One hundred and three subjects (57 healthy controls and 46 presenting aggressive periodontitis) were recruited at the Department of Periodontics, University of Ribeirão Preto Dental School (UNAERP). All subjects were of Brazilian descent. The protocol for the study was reviewed and approved by the Ethics Committee of the UNAERP and written informed consent was obtained from all individuals prior any research activity. All subjects were diagnosed as described above for genetic analysis.

### Gene expression analysis

One biopsy of gingival tissue of each periodontally-healthy subjects (N = 57) were taken from sites that showed no bleeding on probing, probing depth smaller than three millimeters, and clinical attachment loss smaller than one millimeter during surgical procedures due to esthetics, orthodontic or prosthetic reasons. Samples included junctional epithelium, gingival crevicular epithelium and connective gingival tissue. One biopsy of gingival tissue from each aggressive periodontitis patients (N = 46) were taken from the gingival margin to the bottom of the gingival pocket of affected sites, and included junctional epithelium, periodontal pocket epithelium, and connective gingival or granulation tissue. These samples were collected during surgical therapy of the sites that exhibited persistent bleeding on probing and increased probing depth three to four weeks after the basic periodontal therapy (non-responsive sites), as previously described [41]. The extraction of total RNA from periodontal tissue samples was performed with Trizol reagent (Invitrogen, Carlsbad, CA, USA), and the cDNA synthesis was accomplished as previously described [41]. Real-Time-PCR mRNA experiments were performed in a MiniOpticon system (BioRad, Hercules, CA, USA), using SybrGreen MasterMix (Invitrogen, Carlsbad, CA, USA), using 2.5 ng of cDNA in each reaction and primers previously described [41]. Calculations for determining the relative levels of gene expression were made from triplicate measurements of the target gene, with normalization to *β-actin* in the sample, using the cycle threshold (Ct) method and the 2^ΔΔct^ equation, as previously detailed [41].

### Bacterial DNA quantification

In order to allow the detection of *Porphyromonas gingivalis*, *Tannerella forsythia*, *Treponema denticola*, and *Aggregatibacter actinomycetemcomitans*, periodontal crevice/pocket biofilm samples were collected with sterile paper point ISO #40 from the same site biopsied previously to the surgical procedure [41]. Bacterial DNAs were extracted from plaque samples using the DNA Purification System (Promega, Madison, WI, USA). RealTime-PCR mRNA or DNA analyses were performed in a MiniOpticon system (BioRad, Hercules, CA, USA), using SybrGreen MasterMix (Invitrogen, Carlsbad, CA, USA), using 5 ng of DNA in each reaction and the primers previously described [41]. The positivity to bacteria detection and the bacterial counts in each sample were determined based on the comparison with a standard curve comprised by specific bacterial DNA (10^9^ to 10^−2^ bacteria) and negative controls [41]. The sensibility range of bacteria detection and quantification of our real time-PCR technique was of 10^1^ to 10^8^ bacteria to each of the four periodontal pathogens tested.

### Statistical analysis

Calculations of linkage disequilibrium were computed with the Graphical Overview of Linkage Disequilibrium (GOLD) software [42] for both the squared correlation coefficient (*r^2^*) and *Lewontin's* standardized disequilibrium coefficient (*D'*). The program Rutgers Map Interpolator (www.compgen.rutgers.edu/map-interpolator/) was used to convert the physical position of the 14 markers from base pairs to centiMorgans. Non-parametric linkage analysis was performed with the program Merlin [43], [44]. Alleles and haplotypes were tested for association with aggressive periodontitis with the programs Family-Based Association Test (FBAT) [45], [46] and PLINK version 1.05 [47]. To generate odds ratios, the most common allele was used as reference. In the analysis, only probands and relatives with aggressive periodontitis were considered as affected individuals, while relatives who could not be definitely diagnosed with aggressive periodontitis were considered as unaffected individuals (including healthy individuals and individuals with chronic periodontitis). Data was analyzed with and without the family recruited in the Guarulhos University.

Analyses regarding gene expression were performed with t test or by ANOVA, followed by Tukey's test. Multiple logistic and linear regression analyses were performed to evaluate possible associations between the expression of *FAM5C* and inflammatory/immunological and microbial factors. Values of p<0.05 were considered statistically significant.

### Follow up experiments after preliminary results

#### Increasing genotyping density

After the first genotyping results and association analysis, seven additional markers were chosen in the proximity of the rs1342913 marker. The same criteria described previously were used to select these additional markers (Table 2). Genotypes were generated and analyzed as described above.

#### *FAM5C* sequencing

The coding regions, including the exon-intron boundaries of *FAM5C*, were sequenced in eleven unrelated individuals carrying two copies of the haplotype A–G of rs1935881-rs1342913 (nine diagnosed with aggressive periodontitis and two unaffected relatives – Figure S1). As a positive control for good DNA quality, one sample from the Centre D'Étude du Polymorphisme Humain - Fondation Jean Dausset (obtained through Coriell Institute for Medical Research, Camden, NJ, USA) was also sequenced. This sample originated from an anonymous healthy individual. The FASTA sequences of *FAM5C* exons were obtained based on data from the Ensemble Genome Browser (www.ensembl.org). Primer3 (version 0.4.0) (www.primer3.sourceforge.net) was used to design primers covering each exon and exon-intron boundary. *FAM5C* has 8 exons (Figure S3). Primer sequences and polymerase chain reaction conditions are available as Supporting Document (Table S1).

Since no etiologic variants were identified in *FAM5C* coding regions, five highly conserved *FAM5C* intronic sequences were identified in the University of California Santa Cruz Genome Bioinformatics database (www.genome.ucsc.edu) and sequenced (Table S2). Two single nucleotide variants were identified in the conserved regions. These two variants (Table 2) were genotyped in all samples and data was analyzed as described above.

#### Bioinformatic analysis

The program ENDEAVOUR [49] was used to perform gene prioritization in the selected region based on genes already described in the literature as associated with the target disease. A list of 10 genes previously described [14] as showing evidence of involvement with periodontitis in humans was used. Secondly, we used the program TRANSFAC ® 7.0 Public 2005 (www.gene-regulation.com) in order to assess the likely transcription factors binding to the sites of the variants associated with aggressive periodontitis in this study. Finally, the BLAST function (Basic Local Alignment Search Tool) of NCBI (National Center for Biotechnology Information, www.ncbi.nlm.nih.gov) was used to make sequence comparisons between humans and other species in selected nucleotide sequences.

#### Genome Wide Scan

The family recruited in the Guarulhos University (Figure S2) was not associated with markers in 1q (data not shown) and we decided to investigate if this family, in addition to pedigree 24 (Figure S1), would yield the identification of additional contributing loci to aggressive periodontitis, since we previously showed that more than one loci may contribute to the disease [12]. Genome wide genotyping was performed with the GeneChip 500K arrays (Affymetrix, Santa Clara, CA, USA) at the Genomics and Proteomics Core Laboratories, University of Pittsburgh. In brief, two aliquots of 250 ng of DNA each are digested with *Nsp*I and *Sty*I, respectively, an adaptor is ligated and molecules are then fragmented and labeled. At this stage each enzyme preparation is hybridized to the corresponding array. Samples were processed in 96-well plate format; each plate carried a positive and a negative control, up to the hybridization step. A total of 443,816 markers were genotyped. Data was analyzed using the PLINK software.

## Supporting Information

Figure S1 Families recruited in Rio de Janeiro, Brazil. Black color indicates affected individuals. White color indicates unaffected individuals. Arrows indicate proband. Blue color indicates individuals who could not be examined.
(3.99 MB TIF)

Figure S2 Family recruited in the Guarulhos University, Brazil. Black color indicates affected individuals. White color indicates unaffected individuals. Arrow indicates proband.
(0.66 MB TIF)

Figure S3 FAM5C localization in chromosome 1q. Schematic representation of chromosome 1 (top). In the middle is the linkage disequilibrium plot generated for chromosomal region 1q31 including the FAM5C gene. Below is the schematic representation of the FAM5C gene: boxes represent exons, lines connecting boxes are introns. Blue boxes represent untranslated regions and red boxes represent coding regions. The horizontal arrow (bottom) indicates direction of gene.
(0.57 MB TIF)

Table S1 FAM5C primer sequences and polymerase chain reaction (PCR) conditions.
(0.06 MB DOC)

Table S2 FAM5C highly conserved intronic region primer sequences and polymerase chain reaction (PCR) conditions.
(0.04 MB DOC)

Table S3 Association* results between aggressive periodontitis and genetic variation in 1q24.2-1q31.3. *Family-Based Association Test (FBAT).
(0.08 MB DOC)

Table S4 Linkage disequilibrium between markers genotyped in the study. * r2 is above diagonal; D' is below diagonal.
(0.05 MB PDF)

Table S5 Hsplotype results for four-marker windows across the region studied. * f indicates haplotype frequencies.
(0.07 MB PDF)

Table S6 Predicted transcription binding sites for studied markers.
(0.06 MB DOC)
