# Supplementary figures and images for: FAM5C Contributes to Aggressive Periodontitis
Source: PLoS One. 2010 Apr 7;5(4):e10053. doi: 10.1371/journal.pone.0010053 (PMC2850931; doi:10.1371/journal.pone.0010053)

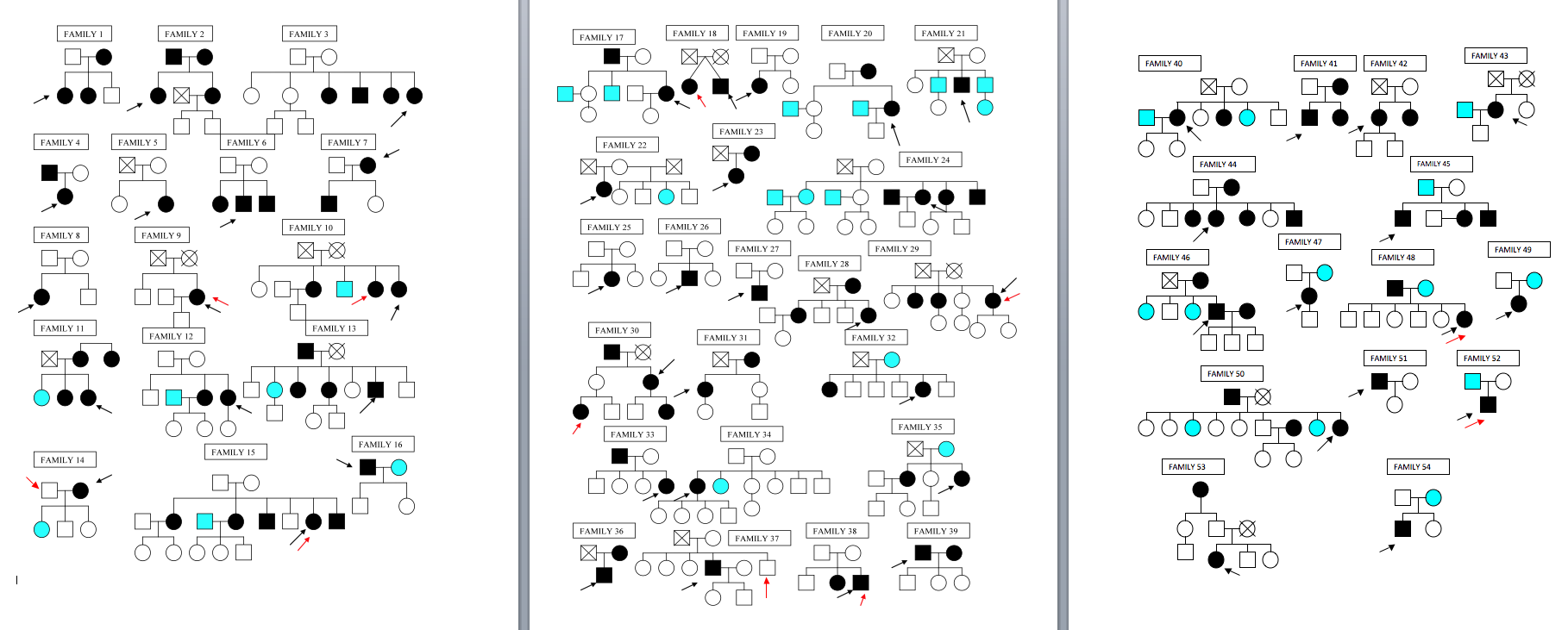

Supplement: Figure S1 — Families recruited in Rio de Janeiro, Brazil. Black color indicates affected individuals. White color indicates unaffected individuals. Arrows indicate proband. Blue color indicates individuals who could not be examined. (3.99 MB TIF) [file pone.0010053.s001.tif]

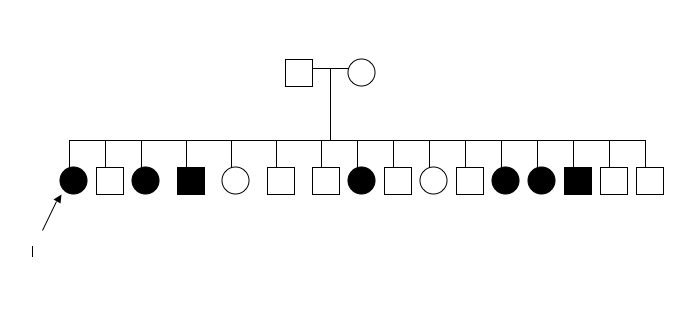

Supplement: Figure S2 — Family recruited in the Guarulhos University, Brazil. Black color indicates affected individuals. White color indicates unaffected individuals. Arrow indicates proband. (0.66 MB TIF) [file pone.0010053.s002.tif]

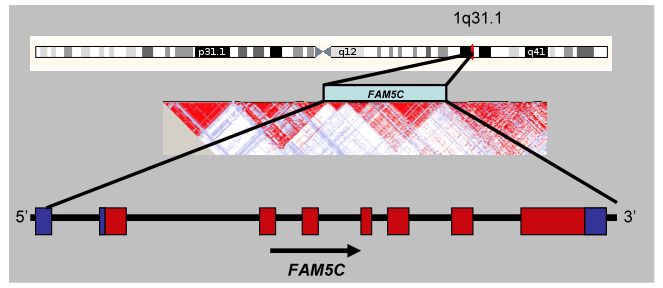

Supplement: Figure S3 — FAM5C localization in chromosome 1q. Schematic representation of chromosome 1 (top). In the middle is the linkage disequilibrium plot generated for chromosomal region 1q31 including the FAM5C gene. Below is the schematic representation of the FAM5C gene: boxes represent exons, lines connecting boxes are introns. Blue boxes represent untranslated regions and red boxes represent coding regions. The horizontal arrow (bottom) indicates direction of gene. (0.57 MB TIF) [file pone.0010053.s003.tif]
