# Supplementary material for: FAM5C Contributes to Aggressive Periodontitis
Source: PLoS One. 2010 Apr 7;5(4):e10053. doi: 10.1371/journal.pone.0010053 (PMC2850931; doi:10.1371/journal.pone.0010053)
Supplement: Table S1 — FAM5C primer sequences and polymerase chain reaction (PCR) conditions. (0.06 MB DOC) [file pone.0010053.s004.doc]

***Table S1. FAM5C primer sequences and polymerase chain reaction (PCR) conditions.***

| Exon | Primer direction | Primer sequence | PCR conditions |
| --- | --- | --- | --- |
| 1 | forward | 5'- ATTCTGCTTTCTTGCCTTGG -3' |  |
|  | reverse | 5' -AATGAAATACTTCAACAAAACAGAGC -3' | 94^0^C for 5 minutes, 35 cycles (94^0^C for 30 seconds, 59^0^C for 30 seconds and 72^0^C for 30 seconds), 72^0^C for 7 minutes |
| 2 | forward | 5'- GCCACCTCAACACCTTAACTG -3' |  |
|  | reverse | 5'- ATTGCTTTCCCTTTGCTTCA-3' | 94^0^C for 5 minutes, 35 cycles (94^0^C for 30 seconds, 59^0^C for 30 seconds and 72^0^C for 30 seconds), 72^0^C for 7 minutes |
| 3 | forward | 5'- GACCCCAGCTACCAAGTGAA -3' |  |
|  | reverse | 5'- GGCACAAATAGGTTCAAAGAGC -3' | 94^0^C for 5 minutes, 35 cycles (94^0^C for 30 seconds, 59^0^C for 30 seconds and 72^0^C for 30 seconds), 72^0^C for 7 minutes |
| 4 | forward | 5'- AGACCTTTCCACCTGCAGATAA -3' |  |
|  | reverse | 5'- TGATAATTTTAGCGTAAACTCATTCTT -3' | 94^0^C for 5 minutes, 35 cycles (94^0^C for 30 seconds, 59^0^C for 30 seconds and 72^0^C for 30 seconds), 72^0^C for 7 minutes |
| 5 | forward | 5'- CAGTGAACCAAAAGGACATTG -3' |  |
|  | reverse | 5'- TGGAAAAGAAATCACGACTGG -3' | 94^0^C for 5 minutes, 35 cycles (94^0^C for 30 seconds, 59^0^C for 30 seconds and 72^0^C for 30 seconds), 72^0^C for 7 minutes |
| 6 | forward | 5'- TGTTAATTTCTTTGTTTGTTCTTCAA -3' |  |
|  | reverse | 5'- AAAAAGGACAAATTAGCCATTTCAA -3' | 94^0^C for 5 minutes, 35 cycles (94^0^C for 30 seconds, 59^0^C for 30 seconds and 72^0^C for 30 seconds), 72^0^C for 7 minutes |
| 7 | forward | 5'- AGTGTTGAATTTTGACATTTTTCAG -3' |  |
|  | reverse | 5'- CTCACTTTTTCGGCAATAAACTT-3' | 94^0^C for 5 minutes, 35 cycles (94^0^C for 30 seconds, 60^0^C for 30 seconds and 72^0^C for 30 seconds), 72^0^C for 7 minutes |
| 8(1) | forward | 5'- TGTAGATTCGTTCTGTTTTGTATTTC -3' |  |
|  | reverse | 5'- TGGACTCGGCGAACTTCAG -3' | 94^0^C for 5 minutes, 35 cycles (94^0^C for 30 seconds, 59^0^C for 30 seconds and 72^0^C for 30 seconds), 72^0^C for 7 minutes |
| 8(2) | forward | 5'- ACACCGGCTACATGCTCAG -3' |  |
|  | reverse | 5'- CAAATCTGTAAAGAGAGACCCAAA -3' | 94^0^C for 5 minutes, 35 cycles (94^0^C for 30 seconds, 59^0^C for 30 seconds and 72^0^C for 30 seconds), 72^0^C for 7 minutes |
| 8(3) | forward | 5'- CAAGTCAAGTCTGGTCCATATGAT -3' |  |
|  | reverse | 5'- GGACCATTGGACTTGATGC -3' | 94^0^C for 5 minutes, 35 cycles (94^0^C for 30 seconds, 59^0^C for 30 seconds and 72^0^C for 30 seconds), 72^0^C for 7 minutes |
| 8(4) | forward | 5'- TTTTGAGACAGTACACATCTACCTGA -3' |  |
|  | reverse | 5'- GACCAGGTGGGGAGAGTTTA-3' | 94^0^C for 5 minutes, 35 cycles (94^0^C for 30 seconds, 59^0^C for 30 seconds and 72^0^C for 30 seconds), 72^0^C for 7 minutes |
| 8(5) | forward | 5'- TGCAGCTGGACTACCCCTAT-3' |  |
|  | reverse | 5'- CATGTGTGTAAATTGCCATCC-3' | 94^0^C for 5 minutes, 35 cycles (94^0^C for 30 seconds, 59^0^C for 30 seconds and 72^0^C for 30 seconds), 72^0^C for 7 minutes |
| 8(6) | forward | 5'- AGCACAACCCAAAATCTTGAA -3' |  |
|  | reverse | 5'- AAGGAATCCCACAGCATTTC -3' | 94^0^C for 5 minutes, 35 cycles (94^0^C for 30 seconds, 59^0^C for 30 seconds and 72^0^C for 30 seconds), 72^0^C for 7 minutes |
