# Supplementary material for: FAM5C Contributes to Aggressive Periodontitis
Source: PLoS One. 2010 Apr 7;5(4):e10053. doi: 10.1371/journal.pone.0010053 (PMC2850931; doi:10.1371/journal.pone.0010053)
Supplement: Table S2 — FAM5C highly conserved intronic region primer sequences and polymerase chain reaction (PCR) conditions. (0.04 MB DOC) [file pone.0010053.s005.doc]

**Table S2. *FAM5C* highly conserved intronic region primer sequences and polymerase chain reaction (PCR) conditions.**

| Intron | Primer direction | Primer sequence | PCR conditions |
| --- | --- | --- | --- |
| 1-2 | forward | 5'-TTTGTCAAAGCGTTCCCTAT -3' |  |
|  | reverse | 5'- GGAAACTAATCTCCTACTTCATCCTG -3' | 94^0^C for 5 minutes, 35 cycles (94^0^C for 30 seconds, 60^0^C for 30 seconds and 72^0^C for 30 seconds), 72^0^C for 7 minutes |
| 1-2 | forward | 5'- AGAATTTATTATAGTTCTCTGCCATGT-3' |  |
|  | reverse | 5'- TTTTGAACAGCATCTGAAACATT -3' | 94^0^C for 5 minutes, 35 cycles (94^0^C for 30 seconds, 60^0^C for 30 seconds and 72^0^C for 30 seconds), 72^0^C for 7 minutes |
| 1-2 | forward | 5'- AAAAAGTACCCTCAGTGCAACC -3' |  |
|  | reverse | 5'- GCTTAATCCTGATGAGCTTGG -3' | 94^0^C for 5 minutes, 35 cycles (94^0^C for 30 seconds, 60^0^C for 30 seconds and 72^0^C for 30 seconds), 72^0^C for 7 minutes |
| 3-4 | forward | 5'- CACAAGACACAGCTGCATGA-3' |  |
|  | reverse | 5'- TCACAGTTGTTTGAGAATTTTACGA -3' | 94^0^C for 5 minutes, 35 cycles (94^0^C for 30 seconds, 60^0^C for 30 seconds and 72^0^C for 30 seconds), 72^0^C for 7 minutes |
| 6-7 | forward | 5'- CATTGAGCCACTACCCCATAA -3' |  |
|  | reverse | 5'- TTGAGAACCCCAAAAGACTG-3' | 94^0^C for 5 minutes, 35 cycles (94^0^C for 30 seconds, 60^0^C for 30 seconds and 72^0^C for 30 seconds), 72^0^C for 7 minutes |
