# Supplementary material for: FAM5C Contributes to Aggressive Periodontitis
Source: PLoS One. 2010 Apr 7;5(4):e10053. doi: 10.1371/journal.pone.0010053 (PMC2850931; doi:10.1371/journal.pone.0010053)
Supplement: Table S3 — Association* results between aggressive periodontitis and genetic variation in 1q24.2-1q31.3. *Family-Based Association Test (FBAT). (0.08 MB DOC) [file pone.0010053.s006.doc]

**Table S3.** Association* results between aggressive periodontitis and genetic variation in 1q24.2-1q31.3.

| Marker | Allele | Allele Frequency | Empirical Variance | Variance | Z | p-value |
| --- | --- | --- | --- | --- | --- | --- |
| rs366839 | A | 0.552 | 2.800 | 7.430 | 1.027 | 0.30 |
|  | G | 0.448 | -2.800 | 7.430 | -1.027 | 0.30 |
| rs463228 | A | 0.465 | -2.533 | 7.452 | -0.928 | 0.35 |
|  | G | 0.535 | 2.533 | 7.452 | 0.928 | 0.35 |
| rs2208921 | A | 0.415 | 1.417 | 6.952 | 0.537 | 0.59 |
|  | G | 0.585 | -1.417 | 6.952 | -0.537 | 0.59 |
| rs12132519 | A | 0.616 | 0.133 | 6.241 | -0.053 | 0.95 |
|  | G | 0.384 | -0.133 | 6.241 | 0.053 | 0.95 |
| rs1935885 | A | 0.397 | 0.250 | 4.938 | 0.113 | 0.91 |
|  | G | 0.603 | -0.250 | 4.938 | -0.113 | 0.91 |
| rs1935881 | G | 0.187 | -5.417 | 5.980 | -2.215 | **0.03** |
|  | A | 0.813 | 5.417 | 5.980 | 2.215 | **0.03** |
| rs35296429 | - | - | - | - | - | - |
|  | A | - | - | - | - | - |
| rs1053081 | A | 0.989 | 1.000 | 1.000 | 1.000 | 0.31 |
|  | G | 0.011 | -1.000 | 1.000 | -1.000 | 0.31 |
| rs35481069 | G | - | - | - | - | - |
|  | T | - | - | - | - | - |
| rs34739035 | G | - | - | - | - | - |
|  | T | - | - | - | - | - |
| rs34098782 | C | - | - | - | - | - |
|  | T | - | - | - | - | - |
| rs10800889 | A | 0.508 | 2.267 | 5.920 | 0.932 | 0.35 |
|  | G | 0.492 | -2.267 | 5.920 | -0.932 | 0.35 |
| rs1342913 | A | 0.656 | -6.200 | 8.563 | -2.119 | **0.03** |
|  | G | 0.344 | 6.200 | 8.563 | 2.119 | **0.03** |
| rs4633293 | A | 0.410 | 2.333 | 9.111 | 0.773 | 0.43 |
| rs12140456 | G | 0.590 | -2.333 | 9.111 | -0.773 | 0.43 |
|  | C | 0.339 | 2.300 | 9.354 | 0.752 | 0.45 |
| rs61818811  rs1377924 | G | 0.661 | -2.300 | 9.354 | -0.752 | 0.45 |
|  | A | - | - | - | - | - |
|  | C | - | - | - | - | - |
|  | C | 0.561 | 0.167 | 10.794 | 0.051 | 0.95 |
| rs2061018 | G | 0.439 | -0.167 | 10.794 | -0.051 | 0.95 |
|  | A | 0.452 | 2.667 | 10.767 | 0.813 | 0.41 |
| rs7526348 | T | 0.548 | -2.667 | 10.767 | -0.813 | 0.41 |
|  | A | 0.504 | 0.700 | 6.296 | 0.279 | 0.78 |
| rs1175111 | G | 0.496 | -0.700 | 6.296 | -0.279 | 0.78 |
|  | A | 0.487 | -0.917 | 8.259 | -0.319 | 0.74 |
| rs1175152 | G | 0.513 | 0.917 | 8.259 | 0.319 | 0.74 |
|  | A | 0.425 | 2.333 | 8.518 | 0.799 | 0.42 |
|  | G | 0.575 | -2.333 | 8.518 | -0.799 | 0.42 |

*Family-Based Association Test (FBAT).
