# Supplementary material for: FAM5C Contributes to Aggressive Periodontitis
Source: PLoS One. 2010 Apr 7;5(4):e10053. doi: 10.1371/journal.pone.0010053 (PMC2850931; doi:10.1371/journal.pone.0010053)
Supplement: Table S4 — Linkage disequilibrium between markers genotyped in the study. * r2 is above diagonal; D' is below diagonal. (0.05 MB PDF) [file pone.0010053.s007.pdf]

**Table S4.** Linkage disequilibrium between markers genotyped in the study.

| SNP | rs366839 | rs463228 | rs2208921 | rs12132519 | rs1935885 | rs1935881 | rs35296429 | rs1053081 | rs35481069 | rs34739035 | rs34098782 | rs10800889 | rs1342913 | rs4633293 | rs12140456 | rs61818811 | rs1377924 | rs2061018 | rs7526348 | rs1175111 | rs1175152 |
|---|---|---|---|---|---|---|---|---|---|---|---|---|---|---|---|---|---|---|---|---|---|
| rs366839 | _ | 0.957 | 0.515 | 0.356 | 0.043 | 0.033 | 0 | 0.017 | 0 | 0 | 0 | 0.126 | 0.004 | 0.012 | 0.001 | 0 | 0 | 0.002 | 0.004 | 0.014 | 0.051 |
| rs463228 | 1 | _ | 0.466 | 0.316 | 0.04 | 0.023 | 0 | 0.015 | 0 | 0 | 0 | 0.147 | 0 | 0.008 | 0 | 0 | 0 | 0.002 | 0.006 | 0.024 | 0.056 |
| rs2208921 | 1 | 0.93 | _ | 0.644 | 0 | 0.018 | 0 | 0.012 | 0.012 | 0.012 | 0 | 0.041 | 0.016 | 0.006 | 0.005 | 0.012 | 0.006 | 0.001 | 0.005 | 0.03 | 0.004 |
| rs12132519 | 0.908 | 0.829 | 0.855 | _ | 0.019 | 0 | 0 | 0.015 | 0.014 | 0.014 | 0 | 0 | 0.013 | 0.005 | 0.008 | 0.014 | 0 | 0.005 | 0.008 | 0.058 | 0.023 |
| rs1935885 | 0.242 | 0.233 | 0.02 | 0.167 | _ | 0.13 | 0 | 0.02 | 0.02 | 0.02 | 0 | 0.253 | 0.041 | 0.037 | 0.055 | 0.02 | 0.025 | 0.033 | 0.016 | 0.003 | 0 |
| rs1935881 | 0.446 | 0.383 | 0.237 | 0.016 | 1 | _ | 0 | 0.002 | 0.036 | 0.036 | 0 | 0.18 | 0.001 | 0.045 | 0.021 | 0.036 | 0.144 | 0.166 | 0.047 | 0.075 | 0.01 |
| rs35296429 | 0 | 0 | 0 | 0 | 0 | 0 | _ | 0 | 0 | 0 | 0 | 0 | 0 | 0 | 0 | 0 | 0 | 0 | 0 | 0 | 0 |
| rs1053081 | 1 | 1 | 0.997 | 0.999 | 1 | 1 | 0 | _ | 0 | 0 | 0 | 0 | 0.025 | 0.008 | 0.022 | 0 | 0.013 | 0.014 | 0.012 | 0.01 | 0.013 |
| rs35481069 | 0 | 0 | 1 | 0.999 | 1 | 1 | 0 | 1 | _ | 1 | 0 | 0 | 0 | 0.012 | 0.023 | 1 | 0 | 0 | 0.012 | 0.01 | 0 |
| rs34739035 | 0 | 0 | 1 | 0.999 | 1 | 1 | 0 | 1 | 1 | _ | 0 | 0 | 0 | 0.012 | 0.023 | 1 | 0 | 0 | 0.012 | 0.01 | 0 |
| rs34098782 | 0 | 0 | 0 | 0 | 0 | 0 | 0 | 0 | 0 | 0 | _ | 0 | 0 | 0 | 0 | 0 | 0 | 0 | 0 | 0 | 0 |
| rs10800889 | 0.439 | 0.462 | 0.234 | 0 | 0.719 | 0.865 | 0 | 0 | 0 | 0 | 0 | _ | 0.002 | 0.032 | 0 | 0 | 0.009 | 0.01 | 0.002 | 0.001 | 0 |
| rs1342913 | 0.073 | 0.004 | 0.174 | 0.148 | 0.228 | 0.096 | 0 | 1 | 0 | 0 | 0 | 0.063 | _ | 0.301 | 0.805 | 0 | 0.002 | 0 | 0.02 | 0.107 | 0 |
| rs4633293 | 0.164 | 0.125 | 0.092 | 0.098 | 0.309 | 0.363 | 0 | 1 | 0.998 | 0.998 | 0 | 0.201 | 1 | _ | 0.296 | 0.012 | 0.021 | 0.032 | 0.006 | 0.025 | 0 |
| rs12140456 | 0.04 | 0.032 | 0.098 | 0.112 | 0.246 | 0.434 | 0 | 1 | 1 | 1 | 0 | 0.02 | 0.944 | 0.909 | _ | 0.023 | 0 | 0 | 0.01 | 0.059 | 0 |
| rs61818811 | 0 | 0 | 1 | 0.999 | 1 | 1 | 0 | 1 | 1 | 1 | 0 | 0 | 0 | 0.998 | 1 | _ | 0 | 0 | 0.012 | 0.01 | 0 |
| rs1377924 | 0.014 | 0.011 | 0.093 | 0.01 | 0.202 | 0.838 | 0 | 1 | 0 | 0 | 0 | 0.107 | 0.069 | 0.184 | 0.024 | 0 | _ | 1 | 0.076 | 0.056 | 0.068 |
| rs2061018 | 0.059 | 0.068 | 0.029 | 0.079 | 0.233 | 0.846 | 0 | 1 | 0 | 0 | 0 | 0.102 | 0.034 | 0.207 | 0.007 | 0 | 1 | _ | 0.08 | 0.043 | 0.056 |
| rs7526348 | 0.087 | 0.108 | 0.072 | 0.093 | 0.171 | 0.454 | 0 | 1 | 0.998 | 0.998 | 0 | 0.053 | 0.187 | 0.079 | 0.138 | 0.998 | 0.276 | 0.283 | _ | 0.262 | 0.265 |
| rs1175111 | 0.158 | 0.192 | 0.199 | 0.303 | 0.076 | 0.548 | 0 | 0.97 | 0.969 | 0.969 | 0 | 0.027 | 0.531 | 0.17 | 0.374 | 0.969 | 0.261 | 0.237 | 0.543 | _ | 0.679 |
| rs1175152 | 0.334 | 0.333 | 0.067 | 0.209 | 0.022 | 0.229 | 0 | 0.999 | 0 | 0 | 0 | 0.005 | 0.021 | 0.005 | 0.016 | 0 | 0.266 | 0.236 | 0.547 | 0.949 | _ |

* $r^2$ is above diagonal; *D'* is below diagonal
