# Supplementary material for: FAM5C Contributes to Aggressive Periodontitis
Source: PLoS One. 2010 Apr 7;5(4):e10053. doi: 10.1371/journal.pone.0010053 (PMC2850931; doi:10.1371/journal.pone.0010053)
Supplement: Table S5 — Hsplotype results for four-marker windows across the region studied. * f indicates haplotype frequencies. (0.07 MB PDF) [file pone.0010053.s008.pdf]

Table S5. Hsplotype results for four-marker windows across the region studied.

<table>
<tr><th colspan="22">Aggressive Periodontitis (HBAT)</th></tr>
<tr><th colspan="21">FAM5C</th><th></th></tr>
<tr><th>rs366839
M1</th><th>rs463228
M2</th><th>rs2208921
M3</th><th>rs12132519
M4</th><th>rs1935885
M5</th><th>rs1935881
M6</th><th>rs35296429
M7</th><th>rs1053081
M8</th><th>rs35481069
M9</th><th>rs34739035
M10</th><th>rs34098782
M11</th><th>rs10800889
M12</th><th>rs1342913
M13</th><th>rs4633293
M14</th><th>rs12140456
M15</th><th>rs61818811
M16</th><th>rs1377924
M17</th><th>rs2061018
M18</th><th>rs7526348
M19</th><th>rs1175111
M20</th><th>rs1175152
M21</th><th></th></tr>
<tr><td>O.30</td><td>0.35</td><td>0.60</td><td>0.96</td><td>0.91</td><td>0.03</td><td>-</td><td>0.32</td><td>-</td><td>-</td><td>-</td><td>0.35</td><td>0.03</td><td>0.44</td><td>0.45</td><td>-</td><td>0.96</td><td>0.41</td><td>0.78</td><td>0.75</td><td>0.42</td><td>f</td></tr>
<tr><td colspan="4">0.18</td><td colspan="17"></td><td>0.018</td></tr>
<tr><td></td><td colspan="4">0.17</td><td colspan="16"></td><td>0.186</td></tr>
<tr><td colspan="2"></td><td colspan="4">0.22</td><td colspan="15"></td><td>0.216</td></tr>
<tr><td colspan="3"></td><td colspan="4">0.09</td><td colspan="14"></td><td>0.063</td></tr>
<tr><td colspan="4"></td><td colspan="4">0.08</td><td colspan="13"></td><td>0.151</td></tr>
<tr><td colspan="5"></td><td colspan="4">0.01</td><td colspan="12"></td><td>0.824</td></tr>
<tr><td colspan="6"></td><td colspan="4">0.32</td><td colspan="11"></td><td>0.991</td></tr>
<tr><td colspan="7"></td><td colspan="4">0.32</td><td colspan="10"></td><td>0.990</td></tr>
<tr><td colspan="8"></td><td colspan="4">0.17</td><td colspan="9"></td><td>0.506</td></tr>
<tr><td colspan="9"></td><td colspan="4">0.01</td><td colspan="8"></td><td>0.220</td></tr>
<tr><td colspan="10"></td><td colspan="4">0.04</td><td colspan="7"></td><td>0.196</td></tr>
<tr><td colspan="11"></td><td colspan="4">0.03</td><td colspan="6"></td><td>0.205</td></tr>
<tr><td colspan="12"></td><td colspan="4">0.06</td><td colspan="5"></td><td>0.247</td></tr>
<tr><td colspan="13"></td><td colspan="4">0.22</td><td colspan="4"></td><td>0.156</td></tr>
<tr><td colspan="14"></td><td colspan="4">0.61</td><td colspan="3"></td><td>0.378</td></tr>
<tr><td colspan="15"></td><td colspan="4">0.37</td><td colspan="2"></td><td>0.170</td></tr>
<tr><td colspan="16"></td><td colspan="4">0.17</td><td></td><td>0.124</td></tr>
<tr><td colspan="17"></td><td colspan="4">0.16</td><td>0.009</td></tr>
</table>

* *f* indicates haplotype frequencies.
