# Supplementary material for: FAM5C Contributes to Aggressive Periodontitis
Source: PLoS One. 2010 Apr 7;5(4):e10053. doi: 10.1371/journal.pone.0010053 (PMC2850931; doi:10.1371/journal.pone.0010053)
Supplement: Table S6 — Predicted transcription binding sites for studied markers. (0.06 MB DOC) [file pone.0010053.s009.doc]

**Table S6.** Predicted transcription binding sites for studied markers.

rs1935881

CTTCCCTTTCTACATTCTAGGAACCA**A**TAATCAGCACACTCGGCACAATTAT ancestral allele

seq( 0.. 59) cttccctttctacattctaggaacca**a**taatcagcacactcggcacaattat

Segments:

[3.6.1.0](http://www.gene-regulation.com/pub/databases/transfac/cl/cl.html#3.6.1.0) 7 16 [====TEC1==](http://www.gene-regulation.com/cgi-bin/pub/programs/alibaba2/getmat.cgi?seg=3.6.1.0&file=seq_257.out&left=7&right=16&seq=seq_257#focus)

[3.4.1.0](http://www.gene-regulation.com/pub/databases/transfac/cl/cl.html#3.4.1.0) 14 23 [====HSTF==](http://www.gene-regulation.com/cgi-bin/pub/programs/alibaba2/getmat.cgi?seg=3.4.1.0&file=seq_257.out&left=14&right=23&seq=seq_257#focus)

[1.1.3.0](http://www.gene-regulation.com/pub/databases/transfac/cl/cl.html#1.1.3.0) 20 29 **[=C/EBPalp=](http://www.gene-regulation.com/cgi-bin/pub/programs/alibaba2/getmat.cgi?seg=1.1.3.0&file=seq_257.out&left=20&right=29&seq=seq_257#focus)**

[3.1.2.2](http://www.gene-regulation.com/pub/databases/transfac/cl/cl.html#3.1.2.2) 26 35 **[===Oct-1==](http://www.gene-regulation.com/cgi-bin/pub/programs/alibaba2/getmat.cgi?seg=3.1.2.2&file=seq_257.out&left=26&right=35&seq=seq_257#focus)**

[1.3.1.2](http://www.gene-regulation.com/pub/databases/transfac/cl/cl.html#1.3.1.2) 31 40 [====USF===](http://www.gene-regulation.com/cgi-bin/pub/programs/alibaba2/getmat.cgi?seg=1.3.1.2&file=seq_257.out&left=31&right=40&seq=seq_257#focus)

[3.1.2.1](http://www.gene-regulation.com/pub/databases/transfac/cl/cl.html#3.1.2.1) 46 55 [===Pit-1==](http://www.gene-regulation.com/cgi-bin/pub/programs/alibaba2/getmat.cgi?seg=3.1.2.1&file=seq_257.out&left=46&right=55&seq=seq_257#focus)

CTTCCCTTTCTACATTCTAGGAACCA**G**TAATCAGCACACTCGGCACAATTAT mutant allele

seq( 0.. 59) cttccctttctacattctaggaacca**g**taatcagcacactcggcacaattat

Segments:

[3.6.1.0](http://www.gene-regulation.com/pub/databases/transfac/cl/cl.html#3.6.1.0) 7 16 [====TEC1==](http://www.gene-regulation.com/cgi-bin/pub/programs/alibaba2/getmat.cgi?seg=3.6.1.0&file=seq_258.out&left=7&right=16&seq=seq_258#focus)

[3.4.1.0](http://www.gene-regulation.com/pub/databases/transfac/cl/cl.html#3.4.1.0) 14 23 [====HSTF==](http://www.gene-regulation.com/cgi-bin/pub/programs/alibaba2/getmat.cgi?seg=3.4.1.0&file=seq_258.out&left=14&right=23&seq=seq_258#focus)

[1.3.1.2](http://www.gene-regulation.com/pub/databases/transfac/cl/cl.html#1.3.1.2) 31 40 [====USF===](http://www.gene-regulation.com/cgi-bin/pub/programs/alibaba2/getmat.cgi?seg=1.3.1.2&file=seq_258.out&left=31&right=40&seq=seq_258#focus)

[3.1.2.1](http://www.gene-regulation.com/pub/databases/transfac/cl/cl.html#3.1.2.1) 46 55 [===Pit-1==](http://www.gene-regulation.com/cgi-bin/pub/programs/alibaba2/getmat.cgi?seg=3.1.2.1&file=seq_258.out&left=46&right=55&seq=seq_258#focus)

* *TEC1, HSTF, C/EBPalp, Oct-1, USF* e *Pit-1* are the transcription factors in the binding-sites of rs1935881 when the ancestral allele is considered. When the allele changes, we can observe that 2 transcription factors (*C/EBPalp,*  *Oct-*1) disappear.

rs1342913

GCCTAAAATCAAAAAACTGGAGAAAA**G**ATGACGTGACACTGATCTCCAAGCA ancestral allele

seq( 0.. 59) gcctaaaatcaaaaaactggagaaaa**g**atgacgtgacactgatctccaagca

Segments:

[2.3.1.0](http://www.gene-regulation.com/pub/databases/transfac/cl/cl.html#2.3.1.0) 18 27 **[====Sp1===](http://www.gene-regulation.com/cgi-bin/pub/programs/alibaba2/getmat.cgi?seg=2.3.1.0&file=seq_235.out&left=18&right=27&seq=seq_235#focus)**

[1.1.1.6](http://www.gene-regulation.com/pub/databases/transfac/cl/cl.html#1.1.1.6) 24 37 [=====ATF====](http://www.gene-regulation.com/cgi-bin/pub/programs/alibaba2/getmat.cgi?seg=1.1.1.6&file=seq_235.out&left=24&right=37&seq=seq_235#focus)

[2.3.3.0](http://www.gene-regulation.com/pub/databases/transfac/cl/cl.html#2.3.3.0) 25 34 [=CPE_bind=](http://www.gene-regulation.com/cgi-bin/pub/programs/alibaba2/getmat.cgi?seg=2.3.3.0&file=seq_235.out&left=25&right=34&seq=seq_235#focus)

[1.1.2.0](http://www.gene-regulation.com/pub/databases/transfac/cl/cl.html#1.1.2.0) 28 37 [====CREB==](http://www.gene-regulation.com/cgi-bin/pub/programs/alibaba2/getmat.cgi?seg=1.1.2.0&file=seq_235.out&left=28&right=37&seq=seq_235#focus)

[1.1.3.0](http://www.gene-regulation.com/pub/databases/transfac/cl/cl.html#1.1.3.0) 44 53 [=C/EBPalp=](http://www.gene-regulation.com/cgi-bin/pub/programs/alibaba2/getmat.cgi?seg=1.1.3.0&file=seq_235.out&left=44&right=53&seq=seq_235#focus)

GCCTAAAATCAAAAAACTGGAGAAAA**A**ATGACGTGACACTGATCTCCAAGCA mutant allele

seq( 0.. 59) gcctaaaatcaaaaaactggagaaaa**a**atgacgtgacactgatctccaagca

Segments:

[1.1.3.0](http://www.gene-regulation.com/pub/databases/transfac/cl/cl.html#1.1.3.0) 23 32 **[=C/EBPalp=](http://www.gene-regulation.com/cgi-bin/pub/programs/alibaba2/getmat.cgi?seg=1.1.3.0&file=seq_234.out&left=23&right=32&seq=seq_234#focus)**

[1.1.1.6](http://www.gene-regulation.com/pub/databases/transfac/cl/cl.html#1.1.1.6) 24 33 **[==CRE-BP1=](http://www.gene-regulation.com/cgi-bin/pub/programs/alibaba2/getmat.cgi?seg=1.1.1.6&file=seq_234.out&left=24&right=33&seq=seq_234#focus)**

[2.3.3.0](http://www.gene-regulation.com/pub/databases/transfac/cl/cl.html#2.3.3.0) 24 33 [=CPE_bind=](http://www.gene-regulation.com/cgi-bin/pub/programs/alibaba2/getmat.cgi?seg=2.3.3.0&file=seq_234.out&left=24&right=33&seq=seq_234#focus)

[9.9.51](http://www.gene-regulation.com/cgi-bin/pub/databases/transfac/getTF.cgi?AC=T00051) 24 33 [====ATF===](http://www.gene-regulation.com/cgi-bin/pub/programs/alibaba2/getmat.cgi?seg=9.9.51&file=seq_234.out&left=24&right=33&seq=seq_234#focus)

[1.1.2.0](http://www.gene-regulation.com/pub/databases/transfac/cl/cl.html#1.1.2.0) 28 37 [====CREB==](http://www.gene-regulation.com/cgi-bin/pub/programs/alibaba2/getmat.cgi?seg=1.1.2.0&file=seq_234.out&left=28&right=37&seq=seq_234#focus)

[1.1.3.0](http://www.gene-regulation.com/pub/databases/transfac/cl/cl.html#1.1.3.0) 44 53 [=C/EBPalp=](http://www.gene-regulation.com/cgi-bin/pub/programs/alibaba2/getmat.cgi?seg=1.1.3.0&file=seq_234.out&left=44&right=53&seq=seq_234#focus)

* *Sp1, ATF, CPE bind, CREB* e *C/EBPalp* are the transcription factors in the binding-sites of rs1342913 when the ancestral allele is considered. When we consider the mutant allele, an additional transcription factor (*C/EBPalp*) and a new one (*CRE-*BP1) appear whereas *Sp1* disappears.

rs57694932

CTTTTAAAATTAACAAATGCAACAGTCCAG**A**AAAGTTAATAAAAAAAAGCTTAATCCTGAT ancestral allele

seq( 0.. 59) cttttaaaattaacaaatgcaacagtccag**a**aaagttaataaaaaaaagcttaatcctga

Segments:

[1.1.3.0](http://www.gene-regulation.com/pub/databases/transfac/cl/cl.html#1.1.3.0) 8 17 [===C/EBP==](http://www.gene-regulation.com/cgi-bin/pub/programs/alibaba2/getmat.cgi?seg=1.1.3.0&file=seq_293.out&left=8&right=17&seq=seq_293#focus)

CTTTTAAAATTAACAAATGCAACAGTCCAG**G**AAAGTTAATAAAAAAAAGCTTAATCCTGAT mutant allele

seq( 0.. 59) cttttaaaattaacaaatgcaacagtccag**g**aaagttaataaaaaaaagcttaatcctga

Segments:

[1.1.3.0](http://www.gene-regulation.com/pub/databases/transfac/cl/cl.html#1.1.3.0) 8 17 [===C/EBP==](http://www.gene-regulation.com/cgi-bin/pub/programs/alibaba2/getmat.cgi?seg=1.1.3.0&file=seq_294.out&left=8&right=17&seq=seq_294#focus)

[9.9.428](http://www.gene-regulation.com/cgi-bin/pub/databases/transfac/getTF.cgi?AC=T00428) 29 38 **[===ISGF-3=](http://www.gene-regulation.com/cgi-bin/pub/programs/alibaba2/getmat.cgi?seg=9.9.428&file=seq_294.out&left=29&right=38&seq=seq_294#focus)**

* *C/EBP* is the transcription factor in the binding-sites of rs57694932 when the ancestral allele is considered. When we consider the mutant allele, one new transcription factor (*ISGF-3*) appears.

rs10494634

CATTATGAGAGAATAATTCTTACATGGCAG**A**GAACTATAATAAATTCTAATGTAATGTTC ancestral allele

seq( 0.. 59) cattatgagagaataattcttacatggcagagaactataataaattctaatgtaatgttc

Segments:

[1.1.3.0](http://www.gene-regulation.com/pub/databases/transfac/cl/cl.html#1.1.3.0) 47 56 [=C/EBPalp=](http://www.gene-regulation.com/cgi-bin/pub/programs/alibaba2/getmat.cgi?seg=1.1.3.0&file=seq_295.out&left=47&right=56&seq=seq_295#focus)

[2.1.1.1](http://www.gene-regulation.com/pub/databases/transfac/cl/cl.html#2.1.1.1) 51 60 [=====GR==](http://www.gene-regulation.com/cgi-bin/pub/programs/alibaba2/getmat.cgi?seg=2.1.1.1&file=seq_295.out&left=51&right=60&seq=seq_295#focus)

CATTATGAGAGAATAATTCTTACATGGCAG**T**GAACTATAATAAATTCTAATGTAATGTTC mutant allele

seq( 0.. 59) cattatgagagaataattcttacatggcagtgaactataataaattctaatgtaatgttc

Segments:

[9.9.539](http://www.gene-regulation.com/cgi-bin/pub/databases/transfac/getTF.cgi?AC=T00539) 20 29 **[====NF-1==](http://www.gene-regulation.com/cgi-bin/pub/programs/alibaba2/getmat.cgi?seg=9.9.539&file=seq_296.out&left=20&right=29&seq=seq_296#focus)**

[1.1.3.0](http://www.gene-regulation.com/pub/databases/transfac/cl/cl.html#1.1.3.0) 47 56 [=C/EBPalp=](http://www.gene-regulation.com/cgi-bin/pub/programs/alibaba2/getmat.cgi?seg=1.1.3.0&file=seq_296.out&left=47&right=56&seq=seq_296#focus)

[2.1.1.1](http://www.gene-regulation.com/pub/databases/transfac/cl/cl.html#2.1.1.1) 51 60 [=====GR==](http://www.gene-regulation.com/cgi-bin/pub/programs/alibaba2/getmat.cgi?seg=2.1.1.1&file=seq_296.out&left=51&right=60&seq=seq_296#focus)

* *C/EBPalp* e *GR* are the transcription factors in the binding-sites of rs10494634 when the ancestral allele is considered. When we consider the mutant allele, one new transcription factor (*NF-1*) appears.
